# *Onchocerca lupi* in imported dogs in the UK: implications for animal and public health

**DOI:** 10.1186/s12917-022-03169-9

**Published:** 2022-02-10

**Authors:** John W. McGarry, Rossella Carrozza, Claire Bradley, Maria S. Latrofa, Benjamin L. Makepeace, Domenico Otranto

**Affiliations:** 1 Institute of Infection, Veterinary, and Ecological Sciences, University of Liverpool, Liverpool, UK; 2 Eye Veterinary Clinic Ltd, Leominster, UK; 3 South Devon Referrals, Abbotskerswell, Devon UK; 4 Department of Veterinary Medicine, University of Bari, Valenzano, Italy; 5 Faculty of Veterinary Sciences, Bu-Ali Sina University, Hamedan, Iran

**Keywords:** *Onchocerca lupi*, Importation, Public health, Humans, Dogs, One health

## Abstract

**Background:**

*Onchocerca lupi* is a filarial nematode affecting dogs, and occasionally cats and humans, in continental Europe, North Africa, the Middle East, and the USA. Adult worms are usually found in periocular nodules and enucleation is sometimes required if the infection fails to respond to other treatment options.

**Case presentation:**

Here, we report the presence of *O. lupi* in the UK for the first time. Of two dogs re-homed from continental Europe, one developed an ocular nodule seven years after arrival from Portugal. The conjunctival perilimbal mass in its left eye was surgically removed but despite anthelminthic treatment, a further nodule developed in the same eye six months later. In the second case - a dog imported from Romania 12 months earlier - a perilimbal mass was excised from the left eye and prior anthelminthic treatment was supplemented with oral prednisolone and doxycycline. However, nodules recurred, and the left globe was subsequently enucleated. Conjunctival hyperaemia then appeared in the right eye and neither additional anthelminthic treatment nor removal of worm masses failed to prevent the further development of lesions. Excised adult worms were identified in both cases as *O. lupi* based on morphological characteristics, as well as PCR and sequencing of cytochrome *c* oxidase subunit I and 12S rRNA gene fragments.

**Conclusion:**

*O. lupi* parasitosis can apparently remain cryptic in dogs for several years before any clinical signs manifest. Moreover, the progression of infection can be highly aggressive and recalcitrant to both surgical intervention and anthelminthic treatment. Increasingly, former stray dogs of unknown infection status are entering the UK, raising both veterinary and public health concerns.

## Background

Environmental changes, anthropic behaviour, and animal movements in Europe over the past 20 years have led to increased threats from a range of zoonotic viral, bacterial and protozoal vector-borne diseases [1]. During the same timeframe, there has also been an emergence of conditions caused by various vector-transmitted nematodes, for which dogs and other carnivores act as reservoirs of zoonotic infection [1–3]. The movement of domestic dogs plays an important role in the epidemiology of this type of vector-borne disease, as seen, for example, with mosquito-transmitted *Dirofilaria* spp. [3]. The UK Animal and Plant Health Agency recorded approximately 45,000 imported dogs in 2019, and this figure includes an unknown number of former stray animals from European countries, whose numbers have increased year-on-year for the past seven years. With a history of scavenging and exposure to biting disease vectors, these so-called ‘Trojan dogs’ can be harbingers of unfamiliar, pathogenic parasites including several types of vector-borne nematodes [3], for which treatment is not legally required before crossing borders.

An example of one such vector-borne nematode is the emerging canine eye worm *Thelazia callipaeda*, which is now endemic throughout much of Europe and is associated with corresponding cases in humans [4]. The first reports of canine thelaziosis in the UK were recently diagnosed in dogs re-homed from Italy, France and Romania [5], and coincidentally, that same year a case of human disease in an international traveller was registered [6].

Another more pathogenic eye worm, the filaria *Onchocerca lupi*, has spread rapidly in dogs in several European countries, including Hungary, Germany, Portugal, Spain and Romania [7–12]. This vector-borne nematode is also zoonotic, with the first human case described only 10 years ago [13]. Subsequently, up to 18 patients have now been diagnosed [12] in countries where canine onchocerciasis has become endemic, including the Southwest USA, probably following transportation of an infected dog from Europe [14]. Adult *O. lupi* usually localize in the episcleral tissues of infected dogs, and occasionally cats, whilst their microfilariae are located in the skin, particularly of the head. However, animals with patent infections do not always display overt clinical signs. Indeed, in endemic regions of Portugal, ocular nodules rarely form in dogs with mature, microfilariae-positive infections [15]. Here, we describe the first two cases of imported canine onchocerciasis in the UK, which presented with unusual pathogeneses. This raises concerns for animal and public health given the potential for *O. lupi* to establish in the UK through the increasingly popular practice of re-homing dogs from other European countries.

## Case presentations

**Fig. 1:**
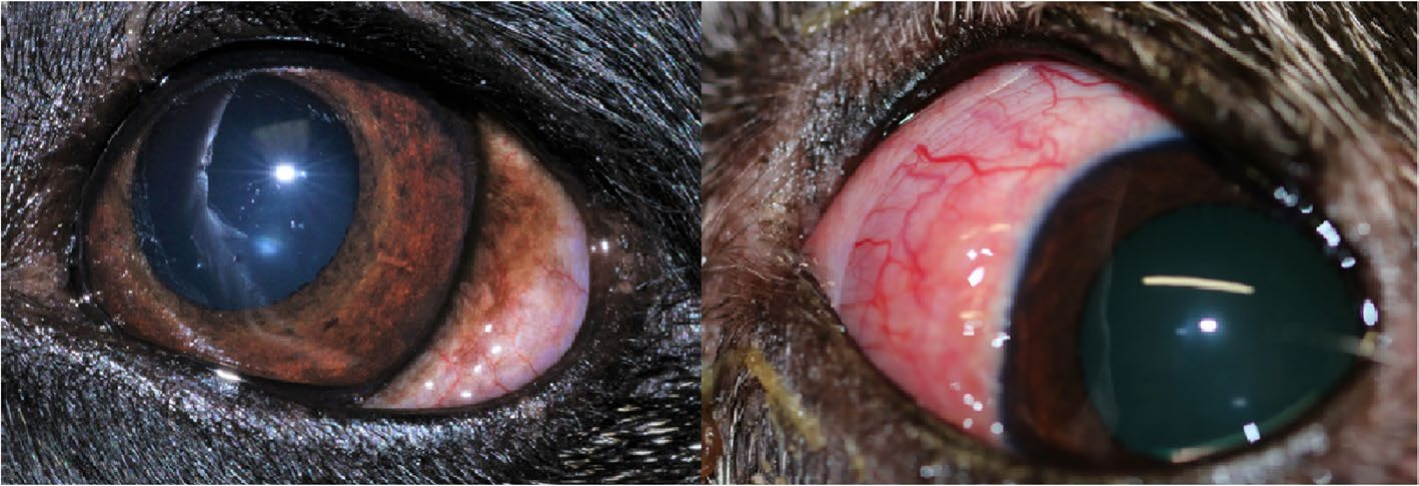
Lesions at presentation. Left, dog originally from Portugal and right, dog imported from Romania

**Fig. 2:**
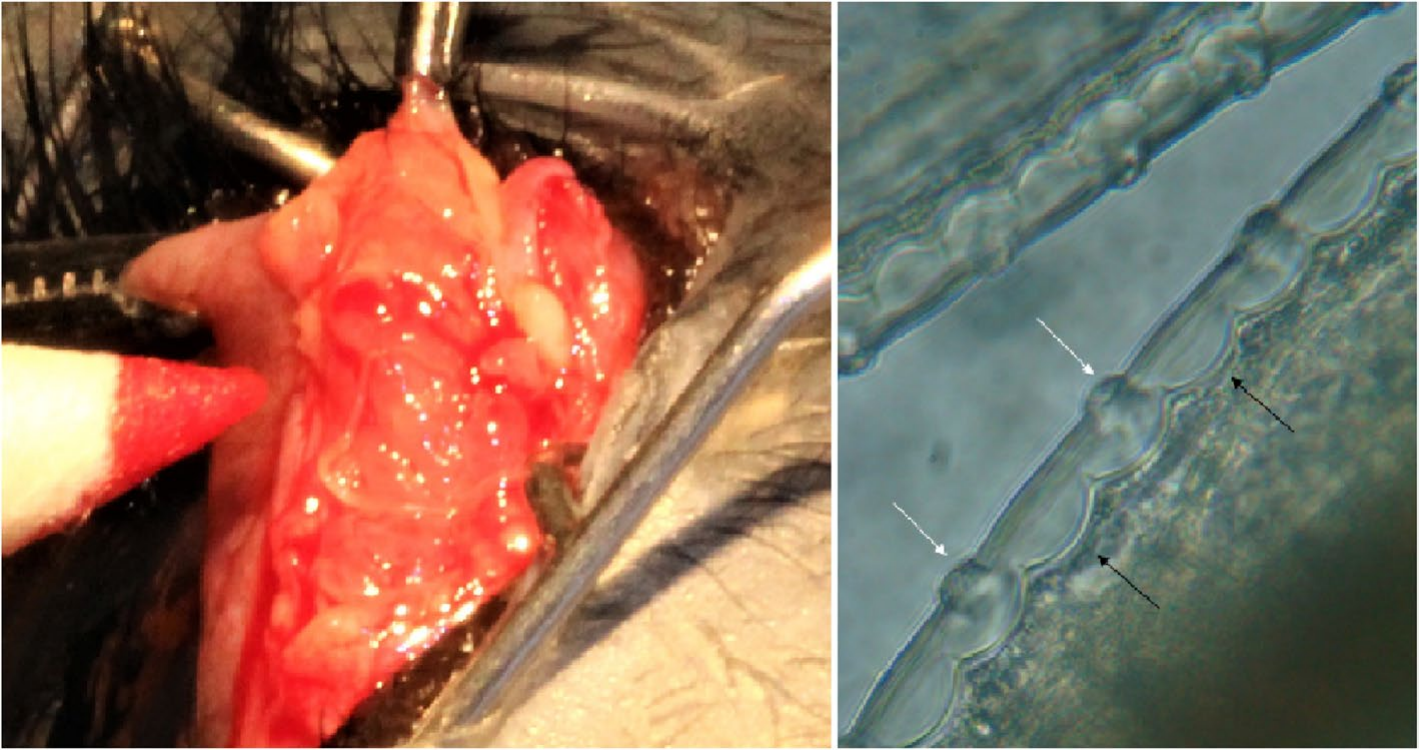
Nodule removed from dog originally from Romania showing cysts and worms; right, characteristic appearance of the cuticle of *Onchocerca lupi*, with arrows showing the typical arrangement of ridges and internal striae

An eight-year-old male crossbreed was investigated in January 2021 for a conjunctival perilimbal mass in the left eye, of two months’ duration (Fig. 1). The dog came from the Algarve region of Portugal aged one year and has remained in the UK ever since. Closely associated with the ventral oblique muscle, the mass was excised in its entirety and revealed multiple small white cysts containing parasite fragments (Fig. 2). Histopathology of the cysts demonstrated parasitic granulomas associated with degenerate and partially mineralised intralesional worms. Treatment included oral doxycycline for three months (targeting the *Wolbachia* symbionts in adult worms) and monthly topical imidacloprid and moxidectin (Advocate®) with alternating monthly low-dose subcutaneous ivermectin (50 μg/kg). Despite this chemotherapy, a further nodule containing worms has since developed in the same eye, six months after removal of the original lesion.

The other dog was a seven-year-old female small crossbreed which entered in the UK as a former stray from Romania in January 2020. Seven months after arrival, and with no history of other travel, a raised tan, perilimbal mass (Fig. 1) was noted in the left eye. Due to progressive exophthalmos, the mass was surgically explored, liberating long, thin nematode fragments, and as in the case above, histology also revealed parasitic granulomas. This dog was already receiving monthly topical imadacloprid and moxidectin (Advocate®) since arriving in the UK, and oral doxycycline and prednisolone therapy was initiated. Three weeks following surgery, additional tan lesions appeared in the same eye and further exploration revealed that the parasites had reached as far as the optic nerve and were strongly associated with the extraocular muscles. Given the extent of disease, the globe was enucleated. One month later, the right eye developed conjunctival hyperaemia. Two injections of melarsomine dihydrochloride were administered 24 h apart, alongside continued oral and topical prednisolone. Over the following three weeks, however, tan nodules appeared which on surgical removal revealed numerous live worms. As of September 2021, further lesions remain.

Nematodes from both cases were morphologically identified as *O. lupi* based on the typical appearance of their cuticle (Fig. 2), which has an arrangement of prominent ridges and internal striae [16]. Identification was also confirmed by sequence analysis of *cox*1 and 12S rRNA gene fragments (15). BLAST analysis (http://blast.ncbi.nlm.nih.gov/ Blast.cgi) of sequences showed a high nucleotide identity with those of *O. lupi* available in GenBank (99.85–100%, *cox*1; 100%, 12S rRNA). Accession numbers deposited in GenBank are MW835250, MW835251, MW829782 and MW829783.

## Discussion and conclusion

*Onchocerca* spp. have unusually long pre-patent periods of up to 18 months [17]. Although autochthonous transmission of canine onchocerciasis in the UK cannot be excluded, it is highly likely that the first dog was infected as a one-year-old in the Algarve region of Portugal, a known focus of transmission [15]. The pre-patent period noted here of seven years is extremely long, but it is comparable to a recent observation in Germany in a dog introduced from Greece, which developed eye problems six years following rescue [18]. Such cases of prolonged parasite and nodule development highlight challenges in case diagnosis and management of *O. lupi*, as well as in monitoring zoonotic disease incursion.

The second case presented an unusually severe pathogenicity, with exophthalmos and disease progression despite appropriate therapy; the invasive nature of nodule growth necessitating globe removal is an exceptional outcome. The re-appearance of worms in the same eye following surgical removal of an existing nodule, as seen in both cases reported here, appears to be a novel finding. In case two, nodule development also occurred in the previously healthy contralateral eye, a matter of weeks after enucleation of the infected eye, and a similar case observation has been previously reported [14]. Together, these findings demonstrate that nematodes can survive undetected until they form overt nodular lesions. In both cases, lesions became prominent, and allowed timely veterinary interventions. However, as already mentioned, not all dogs display overt clinical signs, especially when worms do not develop in the external parts of the ocular apparatus [12]. In undetected covert infections of mature worms, microfilariae will accumulate in the skin for a long time, allowing for potential parasite transmission.

The identity of vectors of this parasite remains unclear, but as for most *Onchocerca* spp., one or more species of *Simulium* (blackflies) may have a role. In the UK, there are at least six species of blackflies recorded as biting both humans and dogs [19], of which *S. reptans* (west of England and Wales) and *S. tuberosum*/*S. variegatum* (north of England and Scotland) are abundant [20]. Considering that the blackfly species composition is similar to that of countries in Europe where *O. lupi* is endemic and assuming they could act as vectors, we hypothesise that local transmission in the UK could occur. An assessment of the suitability of conditions in the UK for parasite circulation by modelling of climate, ecological and other factors is therefore required.

In conclusion, it is apparent that *O. lupi* infection may only become evident in dogs many years following importation. Nodules can be invasive and appear unpredictably with asynchronous development; indeed, our cases show that continual clinical monitoring is required. The popular trend to re-home dogs from *O. lupi*-endemic regions of Europe will increase the risk of autochthonous transmission of this parasite in the UK and it is likely to present a growing problem of One Health concern due to its zoonotic potential.

## Data Availability

For sequence data, accession numbers deposited in GenBank are as follows: MW835250: https://www.ncbi.nlm.nih.gov/nuccore/MW835250 MW835251: https://www.ncbi.nlm.nih.gov/nuccore/MW835251 MW829782: https://www.ncbi.nlm.nih.gov/nuccore/MW829782 MW829783: https://www.ncbi.nlm.nih.gov/nuccore/MW829783

## Abbreviation

*cox*1: cytochrome c oxidase subunit 1
